# Subacute Combined Degeneration Presenting With Prominent Autonomic Symptoms 12 Years After Total Gastrectomy: A Case Report

**DOI:** 10.7759/cureus.84014

**Published:** 2025-05-13

**Authors:** Shinji Morishita

**Affiliations:** 1 Neurology and Rehabilitation, Hanna Chuo Hospital, Nara, JPN

**Keywords:** autonomic dysfunction, gastrectomy, macrocytic anemia, posterior column lesion, sensory ataxia, spastic paraparesis, subacute combined degeneration, vitamin b12 deficiency

## Abstract

Subacute combined degeneration (SCD) is a neurological disorder caused by vitamin B12 deficiency, predominantly affecting the posterior and lateral columns of the spinal cord. While it is frequently associated with pernicious anemia or nutritional deficiency, SCD can also emerge long after gastrectomy due to impaired cobalamin absorption. Although sensory and motor deficits are hallmark features, cases occurring more than a decade post-gastrectomy with prominent, yet reversible, autonomic dysfunction are relatively rare. We report the case of a 48-year-old man who developed SCD approximately 12 years after undergoing total gastrectomy for stage II gastric cancer. Upon admission, he presented with spastic paraparesis, loss of proprioception, and striking autonomic symptoms, including postprandial disturbances resembling late dumping syndrome, bilateral leg edema, and orthostatic hypotension. Laboratory investigations revealed macrocytic anemia, vitamin B12 deficiency, and hyperhomocysteinemia. Spinal MRI demonstrated symmetric T2-weighted hyperintensities localized to the posterior columns of the thoracic cord. Although conventional nerve conduction studies were normal, somatosensory evoked potentials showed significant delays. Treatment with intramuscular vitamin B12 combined with rehabilitative therapy led to substantial recovery of both neurological and autonomic functions, enabling the patient to resume occupational activities within four months. This case highlights the potential reversibility of autonomic dysfunction associated with SCD and emphasizes the importance of early recognition through detailed clinical assessment, particularly when conventional neurophysiological studies fail to detect abnormalities.

## Introduction

Subacute combined degeneration (SCD) is a well-recognized neurological disorder caused by vitamin B12 deficiency. It predominantly affects the posterior and lateral columns of the spinal cord through demyelination, leading to clinical features such as sensory ataxia, paresthesia, and spastic paraparesis [1,2]. Although pernicious anemia and nutritional B12 deficiency are the most frequent causes, SCD may also arise as a delayed complication of gastrectomy due to malabsorption. However, onset more than a decade after surgery is relatively uncommon. Here, we describe a patient who developed SCD approximately 12 years after undergoing total gastrectomy for gastric cancer, underscoring the critical importance of prolonged nutritional surveillance in such individuals [3]. While previous reports have described the onset of SCD within a few years following gastrectomy, such as the case reported by Hwang et al. [3], the latency of more than a decade in our patient is unusual. Furthermore, the prominence and reversibility of autonomic dysfunction in this case add to its uniqueness and clinical value.

Recent literature continues to underscore the neurological implications of vitamin B12 deficiency, particularly its association with SCD and other reversible complications [4-6]. Our patient had not received routine vitamin B12 supplementation following surgery, which may have contributed to the delayed onset of symptoms. Given this background, here, we report a case of SCD that developed 12 years after gastrectomy, presenting with prominent but reversible autonomic dysfunction.

## Case presentation

 A 48-year-old man underwent total gastrectomy with Roux-en-Y reconstruction for stage II gastric cancer in 2010. Postoperatively, he remained recurrence-free and maintained full professional activity as a chef without significant difficulty.

In October 2022, he began experiencing dizziness and palpitations after meals. Concurrently, he developed radiating pain from the buttocks to both lower limbs, progressive gait instability, and bilateral leg edema. As his mobility worsened, he eventually lost the ability to walk unaided, prompting a referral to our department.

Upon admission, his physical measurements were the following: height, 175 cm; weight, 57 kg; and body mass index (BMI), 18.6. Neurological examination revealed no abnormalities in motor strength, sensory modalities, or muscle tone of the upper limbs.

In contrast, the lower limbs exhibited pronounced numbness. Manual muscle testing (MMT) demonstrated mild weakness of the lower extremities (grades 4-5). Muscle tone was elevated, with marked spasticity (Modified Ashworth Scale score of 3), brisk deep tendon reflexes, and bilateral positive Babinski signs. Spasticity was assessed using the Modified Ashworth Scale, a commonly used clinical tool whose interrater reliability has been evaluated in prior studies. Deep tendon reflexes were brisk at the knees but diminished at the ankles.

Sensory examination showed diminished vibration and joint position sense in the lower limbs, accompanied by a positive Romberg sign. Superficial sensations (touch, pain, and temperature) remained relatively intact, with no distinct segmental sensory deficits detected. Motor and sensory functions of the upper limbs, as well as cranial nerve integrity, were preserved.

Laboratory investigations revealed decreased hemoglobin (Hb 7.4 g/dL; reference range, 13.7-16.8) and increased mean corpuscular volume (MCV, 128 fL; reference range, 83.6-98.2), consistent with macrocytic anemia (Table 1). Serum vitamin B12 levels were reduced (203 pg/mL; reference range, 233-914), while homocysteine was markedly elevated (55 μmol/L; reference range, 5-15). Serum vitamin B1 was within normal limits (33.7 ng/mL; reference range, 21.3-81.9).

**Table 1:** Laboratory findings at admission. Key abnormalities include macrocytic anemia, elevated homocysteine, and low-normal vitamin B12 levels. Cerebrospinal fluid (CSF) analysis and thyroid function were within normal limits. Autoantibodies, including PR3-ANCA and MPO-ANCA, were negative. Serum iron and ferritin levels were not measured at admission. Both values were normal at the four-week follow-up. TSH: thyroid-stimulating hormone; PR3: proteinase-3; ANCA: anti-neutrophil cytoplasmic antibody; MPO: myeloperoxidase; FEIA: fluoroenzyme immunoassay

Parameter	Patient's value	Reference range
Hemoglobin	7.4 g/dL	13.7–16.8 g/dL
Mean corpuscular volume (MCV)	128 fL	83.6–98.2 fL
Vitamin B12	203 pg/mL	233–914 pg/mL
Folate (folic acid)	12.0 ng/mL	3.6–12.9 ng/mL
Homocysteine	55 μmol/L	5–15 μmol/L
Vitamin B1	33.7 ng/mL	21.3–81.9 ng/mL
Blood glucose (fasting)	87 mg/dL	73–109 mg/dL
HbA1c	5.40%	4.9%–6.0%
Albumin	4.1 g/dL	4.1–5.1 g/dL
TSH	1.14 μIU/mL	0.500–5.00 μIU/mL
Free T3 (FT3)	1.94 pg/mL	1.68–3.67 pg/mL
Free T4 (FT4)	1.01 ng/dL	0.7–1.48 ng/dL
PR3-ANCA (FEIA)	<0.6 IU/mL	<2.0 IU/mL
MPO-ANCA (FEIA)	<0.2 IU/mL	<3.5 IU/mL
Anti-aquaporin-4 antibody	1.5 U/mL	<3.0 U/mL
CSF cell count	2/μL	0–5/μL
CSF protein	26 mg/dL	15–45 mg/dL
CSF sugar	60 mg/dL	50–75 mg/dL
CSF chloride	124 mEq/L	120–130 mEq/L
Oligoclonal bands	Not detected	Negative

Serum iron and ferritin levels were not assessed at the time of admission; however, both were within normal ranges at the four-week follow-up after initiating vitamin B12 therapy. Blood glucose (87 mg/dL) and HbA1c (5.4%) were unremarkable. Thyroid function tests were normal, and autoantibodies, including PR3-ANCA, MPO-ANCA, and anti-aquaporin-4, were negative.

CSF analysis showed normal cell counts and protein concentrations, with no evidence of oligoclonal IgG bands. Electrophysiological studies of the lower extremities revealed preserved nerve conduction parameters (amplitude, velocity, and latency). Needle electromyography (EMG) of the tibialis anterior, rectus femoris, and triceps surae muscles showed no signs of denervation or neurogenic changes, effectively excluding lower motor neuron involvement.

**Figure 1:**
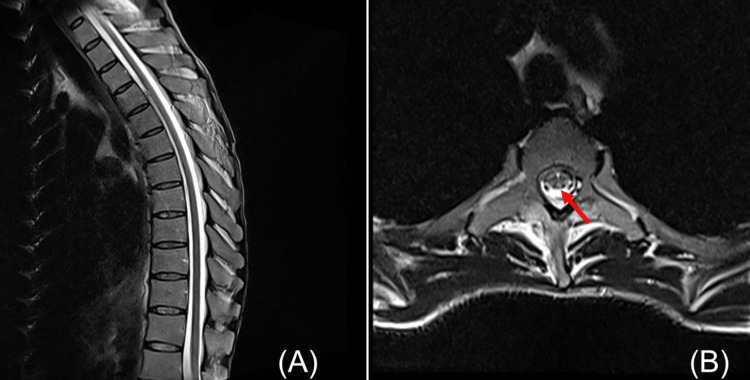
Spinal cord MRI (T2-weighted images). (A) Sagittal T2-weighted image of the thoracic spine showing no apparent abnormal signal intensity. (B) Axial T2-weighted image at the mid-to-lower thoracic level (approximately T8–T9) demonstrating bilateral symmetric high-signal intensity areas along the posterior columns of the spinal cord. Images were obtained using a 1.5-T scanner with standard spinal imaging protocols.

In contrast, somatosensory evoked potentials (SSEP) following right posterior tibial nerve stimulation revealed a mildly prolonged P37 latency (51 ms; normal height-adjusted range, 41-43 ms). Spinal MRI with T2-weighted sequences appeared normal on sagittal views; however, axial sections revealed bilateral, symmetrical T2 hyperintensities along the posterior columns at the T8-T9 vertebral level (Figure 1). Collectively, these findings supported the diagnosis of SCD of the spinal cord secondary to vitamin B12 deficiency, and the patient was admitted for treatment.

At hospitalization, he reported consuming three meals daily and had not experienced any symptoms suggestive of malabsorption, such as chronic diarrhea or unintended weight loss, during the 12-year postoperative period. He was followed by the surgical department for five years after gastrectomy, in accordance with standard postoperative surveillance protocols for gastric cancer in Japan. After this period, no further routine follow-up or vitamin B12 supplementation was provided, and serum vitamin B12 levels were not monitored unless prompted by specific symptoms.

Although there were no chronic gastrointestinal symptoms suggestive of malabsorption, he reported that in the months preceding hospitalization, he had begun experiencing transient postprandial symptoms. Within 30 minutes after eating, he experienced dizziness, sweating, palpitations, abdominal pain, and diarrhea. Additionally, tremors and cold sweats occurred 2-3 hours postprandially. Blood glucose monitoring revealed significant fluctuations: preprandial levels were 90 mg/dL, peaking at 210 mg/dL 30 minutes post-meal, and dropping to 50 mg/dL after 2-3 hours. His diet was adjusted to six smaller meals per day to stabilize glycemic variability, and close clinical monitoring was instituted.

**Figure 2:**
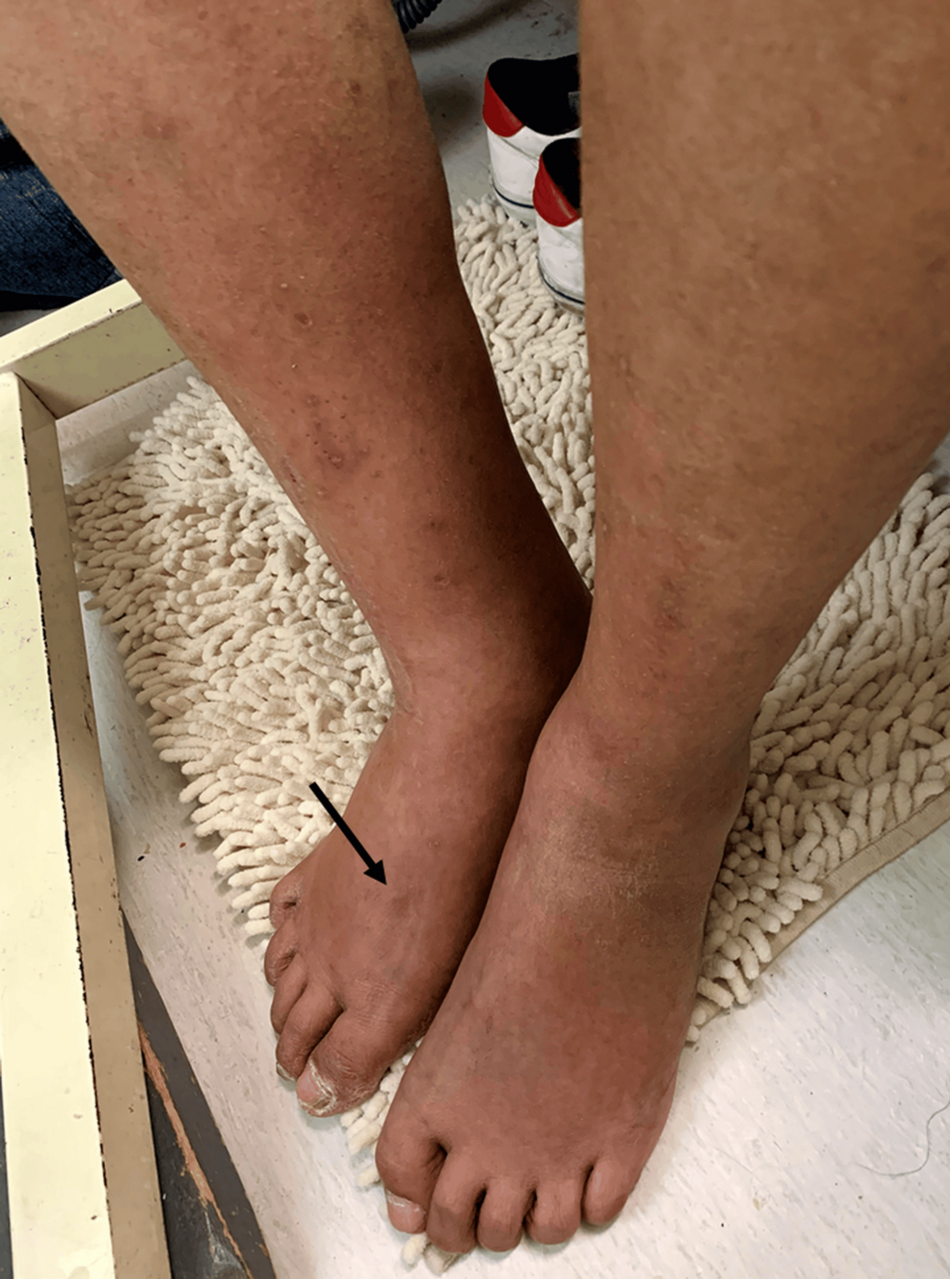
Clinical photograph of the lower extremity. Distal pitting edema is visible, with a marked indentation following applied pressure (indicated by the black arrow).

Physical examination revealed bilateral edematous changes extending from the lower legs to the dorsal aspects of the feet (Figure 2). The affected skin appeared dark reddish, glossy, dry, and scaly. Pitting edema was present (black arrow), with a prolonged pit recovery time (PRT) of 65 seconds. Venous ultrasonography of the lower extremities revealed no evidence of deep vein thrombosis. Laboratory testing showed a normal serum albumin level (4.1 g/dL), and echocardiography excluded cardiac dysfunction. Accordingly, deep vein thrombosis, hypoalbuminemia, and congestive heart failure were ruled out as causes of the edema.

The patient also reported orthostatic symptoms. During an active standing test, systolic blood pressure decreased from 120 mmHg (supine) to 95 mmHg (after three minutes upright), and diastolic pressure dropped from 75 to 60 mmHg, accompanied by a slight heart rate increase from 70 to 73 beats per minute.

Intramuscular methylcobalamin was initiated at a daily dose of 1,500 µg. Clinical improvement followed: after two weeks, orthostatic dizziness and lower limb edema diminished. By the third week, Hb had risen to 10.5 g/dL, and by the fifth week, it had normalized at 12.8 g/dL. His dietary pattern was reverted to three meals per day, with the resolution of postprandial tremors and cold sweats. Two-hour postprandial blood glucose stabilized at approximately 90 mg/dL.

At four weeks, repeat standing tests demonstrated stable systolic pressure (120 mmHg supine, 118 mmHg upright), and orthostatic symptoms had resolved. Edema of the lower extremities had completely subsided.

The patient continued rehabilitation and, within four months, exhibited only mild residual spasticity (Modified Ashworth Scale score of 1+). He successfully regained independent ambulation and was able to resume his profession as a chef.

After discharge, the patient received intramuscular methylcobalamin (1,000 µg three times per week) for three months. He was then transitioned to oral mecobalamin (1,500 µg per day) for lifelong maintenance. To date, no recurrence of macrocytic anemia or neurologic symptoms has been observed.

## Discussion

SCD is a well-recognized neurological disorder caused by vitamin B12 deficiency. It predominantly affects the posterior and lateral columns of the spinal cord through demyelination, leading to clinical features such as sensory ataxia, paresthesia, and spastic paraparesis [1,2]. Although pernicious anemia and nutritional B12 deficiency are the most frequent causes, SCD may also arise as a delayed complication of gastrectomy due to malabsorption. However, onset more than a decade after surgery is relatively uncommon. Here, we describe a patient who developed SCD approximately 12 years after undergoing total gastrectomy for gastric cancer, underscoring the critical importance of prolonged nutritional surveillance in such individuals [3].

Beyond the hallmark neurological deficits, the patient exhibited pronounced autonomic dysfunction at presentation. Notably, symptoms such as postprandial disturbances resembling late dumping syndrome, bilateral lower extremity edema, and orthostatic hypotension were prominent, coinciding with the peak of neurological decline. Encouragingly, the autonomic symptoms fully resolved following vitamin B12 replacement therapy combined with supportive measures, suggesting that the autonomic impairment was reversible.

Although awareness of autonomic involvement in SCD has increased, it remains insufficiently emphasized in standard clinical narratives [4,7,8], contributing to its underrecognition despite a significant impact on patient outcomes. One reason for this is that autonomic fibers are predominantly small-caliber structures-specifically, unmyelinated C fibers and thinly myelinated B fibers-that are poorly assessed by conventional nerve conduction studies [4,9].

Vitamin B12 deficiency can lead to autonomic dysfunction through both demyelination and axonal degeneration of these small-diameter fibers. These fibers are metabolically vulnerable due to their high energy demand and lack of myelin protection. Disruption of sympathetic and parasympathetic pathways may manifest clinically as orthostatic hypotension, postprandial hypotension, gastrointestinal dysmotility, and impaired thermoregulation [4,7]. Consequently, autonomic dysfunction may be present even when electrophysiological evaluations appear normal, highlighting the importance of careful clinical observation.

In real-world clinical practice, access to advanced autonomic testing-such as heart rate variability analysis, the Valsalva maneuver, or sudomotor function testing-is often limited. Furthermore, in this case, the patient expressed a strong preference to prioritize immediate treatment over time-consuming diagnostic evaluations. Bedside assessments, including orthostatic vital sign monitoring and blood glucose measurement, were, therefore, used and provided practical and clinically relevant evidence of autonomic dysfunction. The resolution of these symptoms with vitamin B12 therapy further supported the diagnosis of reversible autonomic neuropathy.

The patient’s postprandial symptoms were consistent with late dumping syndrome, involving vasomotor instability and reactive hypoglycemia. Bilateral, symmetric pitting edema of the lower limbs was observed in the absence of cardiac dysfunction, deep vein thrombosis, or hypoalbuminemia. Orthostatic hypotension was confirmed by an active standing test. Collectively, these findings indicated significant vasomotor autonomic impairment associated with vitamin B12 deficiency, with full recovery following treatment.

Notably, neurological improvement paralleled the resolution of autonomic symptoms. The patient's spastic paraparesis demonstrated considerable recovery, and with consistent rehabilitation, he regained independent mobility and successfully returned to his profession as a chef within four months of hospitalization. This case vividly illustrates the substantial potential for neurological and functional recovery in SCD when timely diagnosis and targeted intervention are instituted.

In summary, this case highlights two pivotal insights: first, SCD can manifest long after gastrectomy, and second, reversible autonomic dysfunction may accompany classical neurological signs, substantially affecting patient morbidity. Vigilance for autonomic symptoms and proactive management-including early diagnosis and sustained vitamin B12 supplementation-are, thus, essential to optimizing outcomes in affected individuals. In this case, the patient remained symptom-free on lifelong oral mecobalamin following initial parenteral therapy, emphasizing the importance of continued maintenance therapy even after apparent recovery.

Furthermore, patients who have undergone total gastrectomy should be provided with long-term nutritional surveillance, including regular screening for vitamin B12 deficiency, in accordance with current recommendations aimed at preventing delayed-onset neurological complications [5].

## Conclusions

SCD may present long after gastrectomy and can involve reversible autonomic dysfunction. Early recognition through detailed clinical assessment, even in the absence of overt neurophysiological abnormalities, is crucial for initiating timely treatment and improving patient outcomes.
